# The impact of training front-line health care workers in improving the yield of scabies diagnosis among children under five in a rural community of West Bengal

**DOI:** 10.1371/journal.pntd.0013739

**Published:** 2025-11-21

**Authors:** Sarika Palepu, Farhad Ahamed, Aparna Palit, Arkapal Bandyopadhyay

**Affiliations:** 1 Department of Community Medicine and Family Medicine, All India Institute of Medical Sciences, Kalyani, India; 2 Department of Dermatology, Venereology and Leprosy, All India Institute of Medical Sciences, Kalyani, India; 3 Department of Pharmacology, All India Institute of Medical Sciences, Kalyani, India; London School of Hygiene and Tropical Medicine, UNITED KINGDOM OF GREAT BRITAIN AND NORTHERN IRELAND

## Abstract

**Introduction:**

Scabies, a parasitic infestation, affects individuals of all age groups. This contagious disease might lead to severe secondary bacterial infections, if left untreated. There is sparse literature available in India, especially in rural areas, focusing on the role of front-line health care workers in determining the prevalence of scabies among children under five. Hence, the primary objective of this study was to assess the impact of training front-line healthcare workers in diagnosing scabies among children under five years in rural areas of West Bengal.

**Methods:**

This community based quasi-experimental (pre-post) study was done in the Saguna Gram Panchayat area. Accredited Social Health Activists (ASHA) (n = 25), the primary point of contact with the community at the grass root level of the health care system in India were assessed with a pre-test. A working training module with imaging templates was prepared. ASHA workers were then trained to identify cases of scabies as per International Alliance for Control of Scabies (IACS) guidelines. Complete enumeration was done and a total of 2,119 children under five years residing in the study area were screened for scabies by ASHA workers. The first author of the study examined all the suspected cases of scabies and 10% of the non-suspect cases as reported by each ASHA worker. A post test was conducted on ASHA workers to assess the efficacy of training.

**Results:**

The mean (±SD) age of ASHA workers was 39.4 (±5.1) years. Most of them were literate up to middle school. There were 2119 children under five years in the study area. Approximately 21 children were suspected of having scabies by ASHA workers. The calculated prevalence of scabies suspect cases was 1%. Most of the family members were unaware of causes, signs, and symptoms of scabies. Data collected by ASHA workers was found to be valid as verified by the first author. There was a significant change in the post-test scores in comparison to pre-test scores of ASHA workers after the training (p-value < 0.05).

**Conclusion:**

The prevalence of scabies in the study area was relatively low. High literacy, good hygienic conditions, absence of malnutrition, and age-appropriate immunization might be the favourable factors for the low prevalence reported. With periodic training, ASHA workers can screen children under five during routine house visits, as seen by their improvement in post-test scores. Accurate estimates of burden can aid in developing standard guidelines for the surveillance and management of this neglected tropical disease.

## Author summary

Scabies, a parasitic skin disease, affects children at varying rates. Left untreated, it can cause serious secondary bacterial infections. The literature concerning the burden of scabies in rural India remains scarce. This study provides valuable data on the prevalence of scabies among children under five in a rural area of West Bengal, India. Our study demonstrates the effectiveness of training ASHA workers to identify suspected cases of scabies. It underscores the need for increased community awareness. This study also provides a model for training community health workers to effectively identify and manage scabies cases. It advocates for integrating scabies screening into routine health check-ups for children as a significant policy decision. It also highlights the need to develop interventions to enhance community awareness about scabies. The findings stimulate further research on improving diagnostic tools and treatment strategies for scabies.

## Introduction

Human scabies, a parasitic infestation by *Sarcoptes Scabei*, is widely prevalent globally. According to Global Burden of Disease survey, in 2021, the prevalence of scabies was 206.6 million and the incidence was 622.5 million cases. It primarily affected children and young adults [1]. In resource-limited settings, about 5 – 50% of children are affected with scabies yearly [2].

According to various studies in India, the burden is estimated to be 13% to 59% [3]. Scabies is a highly contagious yet treatable condition. Transmission from person to person can occur in as short as 15–20 minutes, contributing to high intrafamilial transmission [4]. Besides the distress of clinical manifestations, scabies also impairs the quality of life [5]. According to a study analyzing the global disease burden, scabies accounted for 0.21% of Disability-adjusted Life years in all the countries [6].

The most prominent symptom, i.e., itching, might also lead to secondary bacterial infection due to disruption in skin epithelium. The bacterial infection can further lead to the development of skin sores that, in turn, may cause more severe consequences such as septicemia, bacterial endocarditis, and glomerulonephritis. Scabies has also been documented as a common risk factor for acute kidney damage in 10% of cases in resource-limited settings and rheumatic heart disease in tropical regions [2].

Studies estimating the prevalence of scabies, especially among children under five residing in rural communities are sparse in India. Hence, the burden of the disease is often not known or underestimated. Also, there are also no specific guidelines for diagnosing, reporting, and treatment of scabies, leading to delay in diagnosis and subsequent risk of complications. Accredited Social Health Activists (ASHA), workers are often the primary point of contact for seeking health care in rural areas, and they are stationed at sub-centres or health and wellness centres, the lowest tier of the health care system in India. With fortnightly house visits and regular screening for antenatal, postnatal, and chronic diseases, the frontline health workers have established strong rapport and trust within the community. However, despite their pivotal role, they are not routinely trained to identify and manage neglected tropical diseases (NTDs) like scabies. This gap limits their ability to detect early cases, provide appropriate treatment, and interrupt transmission, underscoring the need for targeted training to strengthen community-based control of scabies. To the best of the authors’ knowledge, there is a paucity of data on the burden of scabies among under-five children in Eastern India.

Therefore, we planned this study with the primary objective to assess the impact of training front-line healthcare workers in estimating the prevalence of suspected scabies cases among children under five years in rural areas of West Bengal and evaluating the effect of the training on the knowledge scores of ASHA workers.

## Materials and methods

### Ethics statement

All parents/caregivers were informed about the purpose of the study and written informed consent was taken. Authors confirm that necessary IRB and/or ethics committee approvals have been obtained. The Institutional Ethics Committee approved the study vide number IEC/AIIMS/Kalyani/Meeting/2022/99.

### Study setting

This quasi-experimental pre-post study was conducted in the Saguna Gram Panchayat area, Kalyani, Nadia district of West Bengal.

### Study population

All ASHA workers associated with the Saguna Health and Wellness Centre were approached for participation in the training and for conducting community-based screening of under-five children. All the children under five years residing in the study area were also the study population.

### Sample size

There were 25 ASHA workers in the study area. All the ASHA workers were the study subjects for the present interventional study. We employed a complete enumeration procedure and interviewed the parents/caregivers of 2119 under-five children residing in the study area.

### Inclusion criteria

All the ASHA workers who provided written informed consent were included in this study. All the children ≤ 5 years of age residing in the study area whose parents/caregivers were willing to provide written informed consent were included in this study.

### Exclusion criteria

Parents/Caregivers who were not present in the residence despite two visits, children who were hospitalized and suffering from any severe or debilitating conditions were planned to be excluded.

As all the parents/caregivers provided consent to participate, no exclusions were made.

### Methodology

The Sarpanch (head of the village in administrative issues) of Saguna Gram Panchayat was approached, and requisite administrative approval was taken. Following this, the first author (SP) visited the Saguna Health and Wellness Centre, where the Community Health Officer (CHO), the in-charge of the centre, was briefed about the objectives of the study. ASHA workers visit the houses of the under-five children fortnightly as part of the field level implementation of various national health programs in India. Database of children under five residing in their designated area is maintained and updated by them during these visits. The first author (PS) ensured that the data were available before starting the study.

Ten structured objective questions focusing on the causative factors, mode of spread, signs and symptoms, treatment modalities and prevention methods to be adopted by family members for scabies were prepared. Pre-testing of ASHA workers was conducted at Saguna HWC and scores were assessed. A training module was then devised (in English) to address the gaps in their knowledge. A translator, proficient in English and native language Bengali has translated the training module to the local language (Bengali). The training module in the native language was back translated to English, and discrepancies with the original module were assessed by another independent translator. ASHA workers were then trained for two consecutive days with the help of audio-visual aids. The classification by International Alliance for Scabies Control (IACS) was adopted in the training module to diagnose scabies in children [7].

**Fig 1:**
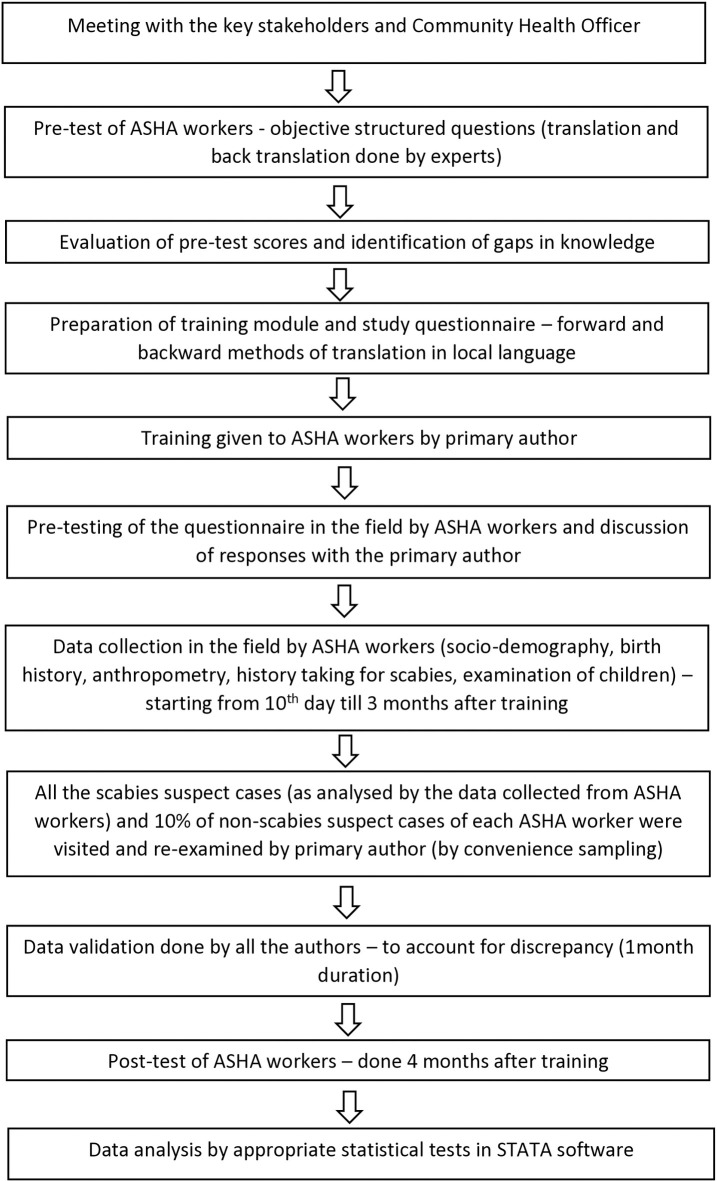
Flow chart of study process.

A structured questionnaire was prepared in English as per the study objectives. Forward and backward translation methods were employed to translate the questionnaire into native language. A question-by-question guide was prepared for the interview schedule to aid in the uniformity of the interview process and address the inter interviewer bias. A one-day training was conducted to explain the contents and method of data collection to ASHA workers. Pre-testing of the questionnaire was done (5 interviews by each ASHA worker), and changes were made accordingly. Data collection was done over a span of 4 months, i.e., from March to July, 2023. Study flow is outlined in Fig 1.

The primary respondents in the study were the mother or the head of the household (based on the criteria of age or earning member of the family) if the child’s mother was unavailable at the time of the visit. ASHA workers interviewed parents/heads of the household about sociodemographic details, morbidity profile, immunization status, and environmental conditions pertinent to scabies. Socio-economic status of the families was categorised based on the BG Prasad (Bhausaheb Gangadhar Prasad) socio-economic evaluation scale [8]. This scale takes into account the per capita monthly income of the family and is valid for use in both urban and rural areas of India. Information regarding the occurrence of scabies in any of the family members was also obtained by them. All the children were then examined for the presence of symptoms (itching) and signs (atypical or typical distribution of lesions, skin burrows) suggestive of scabies and history of contact (as outlined in IACS guidelines) by ASHA workers with the help of training modules and imaging templates provided to them. Also, anthropometric measurements of the children, such as weight, height, and mid upper arm circumference (MUAC), were taken. Data collection was started after 10 days and completed within 3 months of training ASHA workers. To ensure the accuracy of diagnosis, the first author (PS) examined all the scabies suspect and 10% of the cases considered non-scabies by all ASHA workers. Following this, a post-test was conducted on ASHA workers, after four months of training.

### Operational definitions

Severe stunting was defined as low height for age (z-score below -3 Standard deviation) and severe wasting was defined as low weight for height age (z-score below -3 Standard deviation) as per World Health Organisation age and gender appropriate growth charts [9].

### Scabies suspect

The classification was done based on the history given by study respondents (itching and positive contact history as per IACS guidelines or similar complaints in family members) or on physical examination by ASHA workers at the time of visit (presence of skin burrows/papules/vesicles in typical or atypical sites – face, neck, palms and soles etc) [7].

### Statistical analysis

Data entry was done in Microsoft Excel 2013. All the analyses were done in STATA ver.17 (Statistics and Data). Continuous variables were expressed as mean (±SD) and categorical variables as percentages. The difference in pre and post test scores of ASHA workers was assessed with Wilcoxon signed rank test after applying Shapiro Wilk test of normality.

### Patient and Public involvement

It was not appropriate or possible to involve patients or the public in the design, or conduct, or reporting, or dissemination plans of our research.

## Results

There were 25 ASHA workers in the study area. The mean age (SD) of ASHA workers was 39.4 (5.1) years. Most of them were educated till middle school. The duration of their work experience ranged from 8 to 12 years. All of them belonged to rural areas and most of them resided in nuclear families (n = 19, 76%).

Universal sampling was done and in 2042 families, there were 2119 children under five years of age. Most families (n = 1955, 95.7%) had one child under five years of age and in few families (n = 87, 4.3%), there were 2 children. Respondent was mother in 97% (n = 1981), father in 1.2% (n = 25), followed by relatives/other family members in 1.8% (n = 36). The response rate was 100%, and there was no refusal for participation. Socio-demographic characteristics of the family members was taken by the ASHA workers. The mean age of mothers in this study was 27.8 years, educated up to middle school (28%), followed by high school (21%), and most (97%) were homemakers. The mean age of fathers was 34.5 years, 30% were educated up to middle school, and most (99.4%) were employed (Table 1).

**Table 1:** Sociodemographic characteristics of the parents (study respondents).

Sl. No	Variable		Mother (n = 2042)	Father (n = 2042)
1.	Age (in years)	Mean (±SD)	27.8 (±5.26)	34.5 (±5.8)
		Range	16–44	19–52
2.	Education	No formal education	12 (0.6%)	16(0.8%)
		Primary school	245 (12%)	368 (18%)
		Middle school	571 (28%)	612 (30%)
		High school	429 (21%)	347 (17%)
		Intermediate	368 (18%)	286 (14%)
		Graduate/Postgraduate	368 (18%)	368 (18%)
		Professional	49 (2.4%)	45 (2.2%)
3.	Occupational status	Employed	62 (3.0%)	2030 (99.4%)
		Unemployed/Homemaker	1980 (97%)	12 (0.6%)

Most of them were residing in nuclear families (n = 1306, 64%) with a median (IQR) family size of 4 (4–5). Median income of the family was 10,000 Indian rupees (IQR: 7,000 – 12,000). The majority of the families were Hindu by religion (n = 1679, 82.2%), followed by Muslim (n = 312, 15.3%) and Christian (n = 51, 2.5%). As per the BG Prasad scale, about 40% (n = 817) families belonged to the lower middle class, followed by middle (n = 592, 29%), lower (n = 306, 15%), upper middle (n = 245, 12%) and upper (n = 82, 4%).

**Fig 2:**
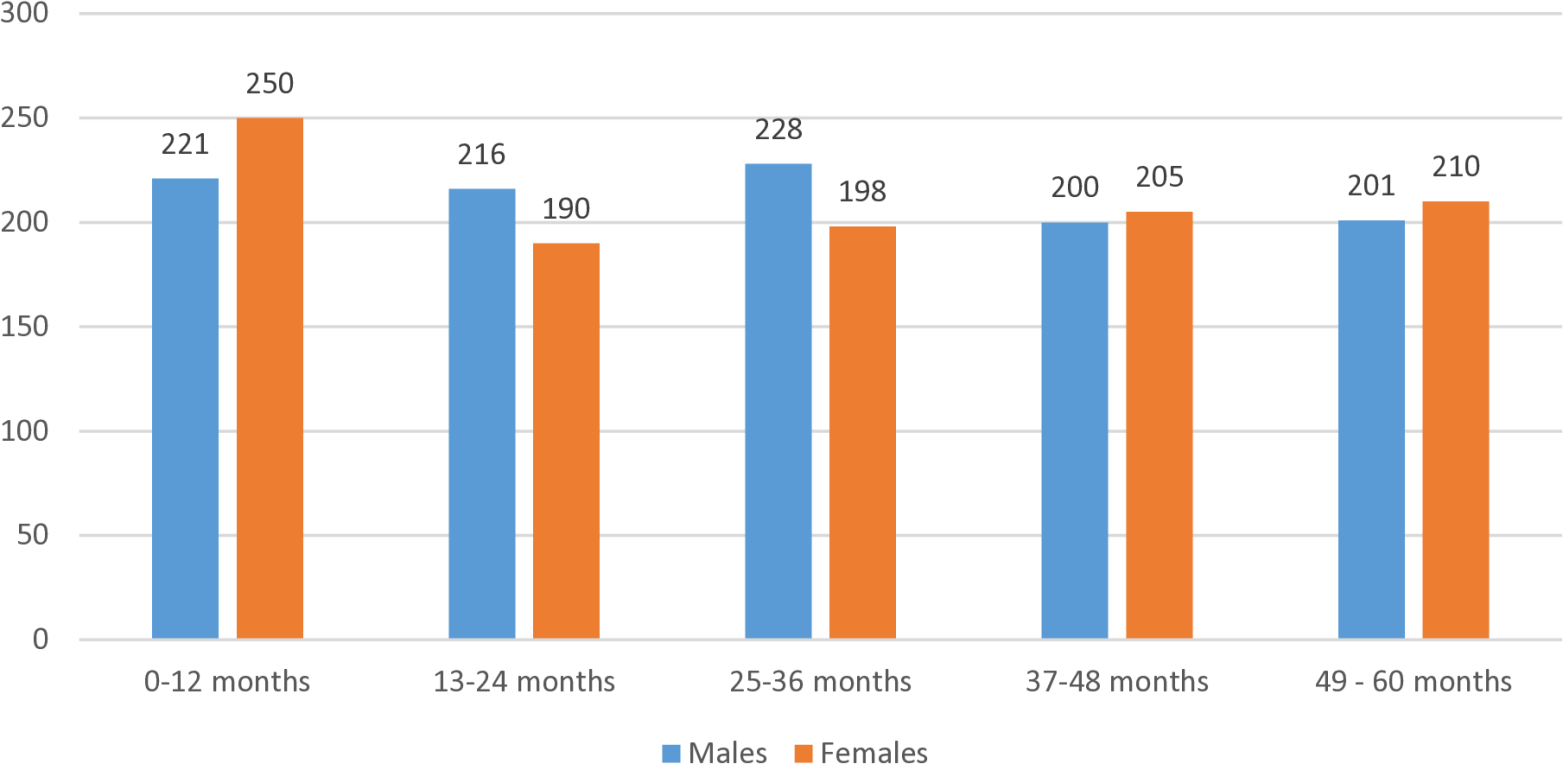
Distribution of under-five children by age and gender.

In the study area, there were about 2119 children under five years. There were 1065 male and 1054 female children in the study area (Fig 2). The child sex ratio was 989 females per 1000 males. The mean age of mothers at the time of childbirth was 25.3 (5.2) years. The mean weight at birth of the children was 2707.6 grams (SD = 458.3). Birth weight significantly differed between males and females (males = 2740 grams, females = 2675 grams, p-value < 0.05). The prevalence of low birth weight (weight below 2500 grams) in the study area was 23% (n = 479). The natal characteristics of the children were also studied to assess the representativeness of the sample and health care seeking behaviour. Majority of the children were born after 37 completed weeks of gestation (97.8%), mostly by lower segment caesarean section (74.3%) and at private health care facilities (57.8%). None of the children were into formal school education at the time of conduct of study.

The mean age of the children at the time of conduct of the study was 30.7 (±18.1) months. About 95% of the children were immunized for age. Only two children were severely stunted, and 4 of them were severely wasted. The anthropometric characteristics of children are tabulated below (Table 2).

**Table 2:** Anthropometric characteristics of the children by age.

Sl. no	Age (in years)	Mean (±SD) weight (in Kgs) (95% CI)	Mean (±SD) height (in cms) (95% CI)
1	<1	7.23 (2.55) (6.79–7.79)	63.46 (0.63) (62.22–64.69)
2	1 – < 2	10.53 (2.35) (10.07–10.99)	77.64 (0.50) (76.65–78.63)
3	2 – < 3	12.57 (1.48) (12.28–12.87)	87.16 (0.61) (85.96–88.37)
4	3 – < 4	13.97 (1.52) (13.67–14.27)	94.01 (0.63) (92.77–95.25)
5	4 – 5	15.60 (1.65) (15.28–15.93)	101.69 (0.65) (100.42–102.95)

As per the data collected by ASHA workers, 21 children had itching and 22 children had positive contact history of scabies. On inspection, only two children had skin burrows. Among the children with itching and positive contact history, five children were already diagnosed with scabies at a healthcare facility at the time of data collection. Only two of the diagnosed cases reported seeking treatment, both of whom accessed care through private healthcare facilities (Table 3).

**Table 3:** Characteristic features of scabies among children under five years of age.

Sl. No	Characteristic	Number (n)
1	Skin burrows	2
2	Typical lesions affecting genitalia (in males)	0
3	Typical lesions in typical distribution	1
4	Atypical lesions or atypical distribution	2
5	Itching	21
6	Positive contact history	22

*Multiple symptoms were present in few children and hence n ≠ 21.

**Fig 3:**
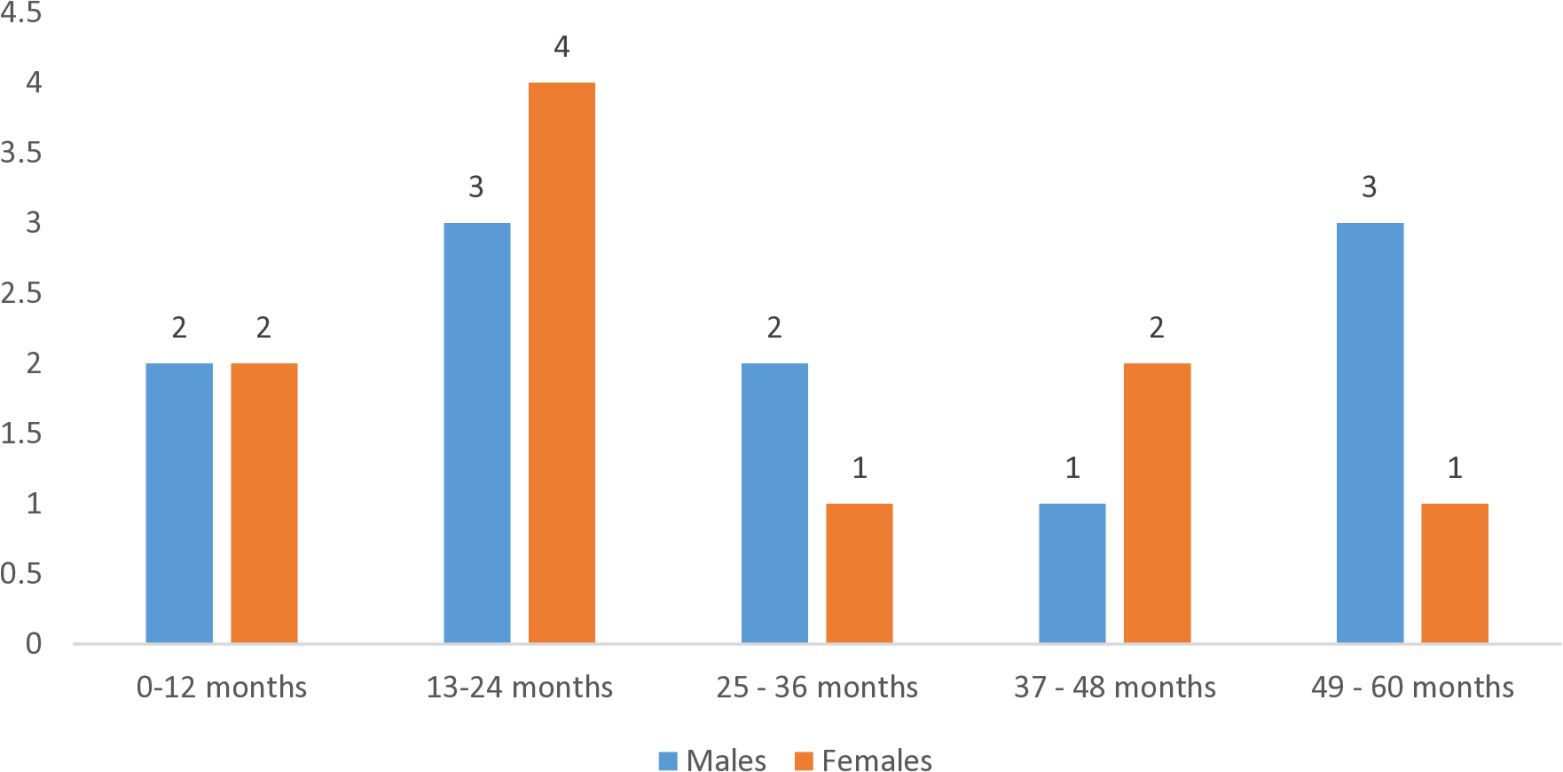
Age and gender-wise distribution of suspected scabies cases.

Age and gender-wise distribution of suspected scabies cases (n = 21, based on itching and positive contact history) is depicted in Fig 3.

A history of scabies diagnosis was reported in only three families within the study area, involving a total of five affected individuals with ages ranging from 18 to 67 years (18, 19, 41, 42, and 67 years). Respondents’ (parents/caregivers) awareness regarding scabies was minimal. Only 20 respondents could acknowledge it as a skin disease. Notably, none could accurately identify the signs or symptoms associated with scabies. In this study, we have also assessed the environmental details of the family to assess the association with scabies. Water was adequately covered in most families (93.3%). None of the families reported sleeping on the floor. Overcrowding was seen in only 20 families (0.9%). There was no history of sharing clothes/linen among family members. All the children had good personal hygiene, as observed by ASHA workers. Mothers reported that the children were bathed regularly.

The first author (SP) then screened all the suspected cases of scabies by house visits. About 10% of the non-suspected cases under each ASHA worker were also independently screened by the first author (PS), and none exhibited symptoms suggestive of scabies. The screening was done by convenient sampling method. The results of the visits were compared with the data collected by ASHA workers by all the authors. No disparity in the study results was seen and the data collected by ASHA workers was valid. Based on the study findings, the prevalence of suspected scabies among children under five years of age was estimated to be 1% (21 children out of 2119 had signs/symptoms and positive contact history) (Table 3).

A post-test of ASHA workers was conducted after 4 months of training. The pre-test mean scores were 2.44 (SD = 1.00. Range: 1 – 4, 95% CI: 2.03 - 2.85) and the post-test mean scores were 7.24 (SD = 1.64, Range: 4 – 9, 95% CI: 6.56 – 7.92). The difference in mean pre-test and post-test scores was evaluated using the Wilcoxon signed rank test. The post-test scores were significantly higher than the pre-test scores. (P-value – 0.00)

The study significantly increased the knowledge of frontline healthcare workers, as evidenced by their accurate diagnosis of suspected scabies cases and the significant increase in post-test scores.

## Discussion

The present study aimed to assess the prevalence of scabies among children under five and their family members. To the best of the researchers’ knowledge, limited research studies were done in Eastern India in this age group. Assessing scabies in this age group holds significance as severe and disabling pruritis in infants can lead to poor feeding [10]. This might initiate a cascade of events leading to malnutrition and increased susceptibility to further infections.

The mean age of mothers in this study was 27.8 years, 28% were educated till middle school, and most were homemakers. The literacy rate in the study is relatively high (99.6%) compared to the national statistics of National Family Health Survey -5 (NFHS) among adult females (65.9%) in rural areas. The mean age of mothers at childbirth was 25.3 years, almost similar to NFHS-5 statistics of 27.6 years [11].

There were about 2119 children under five years of age, and the child sex ratio was 989 females per 1000 males, similar to West Bengal statistics of 973 but higher than the national statistics of 929 [12]. The mean weight at birth was 2707.6g (SD = 458.3). Birth weight significantly differed between males and females (males = 2740g, females = 2675g, p-value < 0.05). The prevalence of low birth weight in the area was 23% (n = 479), higher than the national statistics of 17.29%. Various determinants leading to an increased proportion of low-birth children in the study area must be assessed in greater detail [13].

The prevalence of suspected scabies in this study was 1%. Very few studies have been conducted in this age group to assess scabies. A study done among children 5–14 years old in orphanages in Tamil Nadu showed the prevalence to be 7.2% [14]. Another study done by Kumar et al. showed that 12.57% of children in the age group of 5 – 20 years were having scabies [15]. The varied prevalence might be attributable to the different age groups and study settings. Another study by Behera P et al. in tribal communities showed the prevalence as 10.1% among children under five (as per the dataset provided by authors) [16].

In this study, respondents’ awareness of the cause, signs, and symptoms of scabies was deficient. Despite this, the lower prevalence of scabies in the study area might be attributed to the family’s sanitized living conditions, maintenance of good personal hygiene, not sharing linen/clothes, and the absence of overcrowding and contact with suspect/confirmed scabies cases. Most of the parents were literate and lived in sanitized conditions. Most children were fully immunized and well nourished, decreasing the chances of scabies infection.

A key strength of this study lies in its pioneering approach of training ASHA workers to assess suspected scabies cases through universal community screening, with the findings verified by the primary author (SP). The information collected by ASHA workers was entirely valid, and the post-test scores were significantly higher, demonstrating the effectiveness of the training. However, there might be underreporting of cases as ASHA workers were the primary data collectors in the study. They might have underestimated the cases due to the burden of implementing various national health programs at the field level and because they received only a two-day training session. The primary limitation of this study was that it assessed the knowledge and practice domain of ASHA workers through pre and post-test evaluations at an interval of only four months. Further research could be conducted to evaluate the long-term impact of training on ASHA workers’ ability to effectively identify scabies cases. The study possesses high internal and external validity, as universal screening was conducted for all children residing in the study area.

Routine screening of the community by ASHA workers has the potential to support identifying cases of neglected tropical skin diseases. This will require appropriate credentialing with targeted training using suitable materials in local languages and the introduction of performance-based incentives. Expanding training to also include other neglected tropical skin diseases could potentially enhance community-level surveillance and strengthen public health efforts in rural areas.

## Conclusion

The prevalence of scabies in the study area was relatively low, likely due to good nutritional status, up-to-date vaccinations, good personal hygiene, and favourable household conditions. Despite this, community awareness of scabies was limited. This study demonstrates that rapid, targeted training of ASHA workers can significantly improve their knowledge, facilitate early screening, and support estimation of community-level prevalence. Integrating routine scabies monitoring and community awareness initiatives into national health programs, supported by standardised protocols for community health workers, can offer a practical approach to strengthening surveillance and management of neglected tropical diseases.

## Supporting information

S1 Checklist STROBE checklist [17]. (DOCX)

S1 File Training manual. (DOCX)

S2 File Study questionnaire. (DOCX)
