# Supplementary material for: The impact of training front-line health care workers in improving the yield of scabies diagnosis among children under five in a rural community of West Bengal
Source: PLoS Negl Trop Dis. 2025 Nov 21;19(11):e0013739. doi: 10.1371/journal.pntd.0013739 (PMC12671740; doi:10.1371/journal.pntd.0013739)
Supplement: S1 File — (DOCX) [file pntd.0013739.s002.docx]

**All India Institute of Medical Sciences, Kalyani**

(A Statutory body under aegis of

Ministry of Health and Family Welfare, Govt. of India)

NH - 34 Connector, Basantapur, Saguna, West Bengal – 741245

**Guide book for diagnosing scabies among children by ASHA workers**

**Project Title:** The impact of training front-line health care workers in improving the yield of scabies diagnosis among under five children in a rural community of West Bengal

**Principal Investigator:** Dr. Sarika Palepu (Email Id: [sarika.cmfm@aiimskalyani.edu.in](mailto:sarika.cmfm@aiimskalyani.edu.in))

**Funding agency:** International Alliance for the Control of Scabies, Royal Society of Tropical Medicine and Hygiene

**What is Scabies??**

It is a skin disease caused by infestation of the upper layers of the skin by human itch mite and manifested as follows:

- Itching and a pimple like skin rash (scabies rash) - most commonly seen.
- Severe itching (pruritus), especially at night - the earliest and most common symptom.
- Tiny burrows are often seen in webbing between the fingers, in the skin folds on the wrist, elbow, or knee, and on the penis, breast, or shoulder blades.

May affect much of the body or be limited to: Between the fingers, Wrist, Elbow, Armpit, Penis, Nipple, Waist, Buttocks, Shoulder blades. The head, face, neck, palms, and soles often are involved in infants and very young children.

.
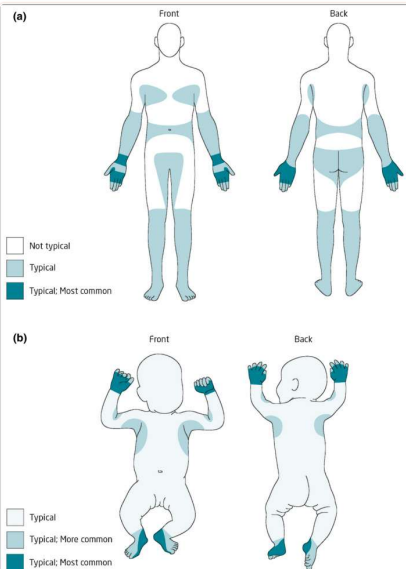


1. Children ≥2 years of age and adults b. Children <2 years of age

**Scabies: Risk Factors, Transmission, Complications, and Prevention**

**What are the risk factors for scabies?**

Over-crowding and poor sanitation favours the spread.

**How does scabies spread?**

Spreads by direct, prolonged, skin-to-skin contact with a person who has scabies. A person infested with scabies can favour spread although he/she does not have any symptoms. If infected for first time – symptoms develop after 2-6 weeks. If infected previously – symptoms can develop in 3-4 days.

**What are the complications of scabies?**

Intense itching can lead to skin sores by scratching. The skin sores if infected with bacteria cause secondary skin infection and also affect kidneys.

**How can scabies be prevented?**

Avoid direct skin-to-skin contact and clothing or bedding used by an infested person. Simultaneous treatment usually is recommended for members of the same household and other potentially exposed persons Bedding and clothing used 3 days before treatment should be washed and dried using hot water and sun-dried. Disinfection by storing in a closed plastic bag for several days to a week can also be followed (scabies mite survives only for 2-3 days’ outside human body). Children can return to child care or school the day after treatment.

**How to identify a child with scabies?**

**Instructions:** *Please look for the below shown lesions in children from head to toe.*

1. **Child with scabies rash over face and body**

**
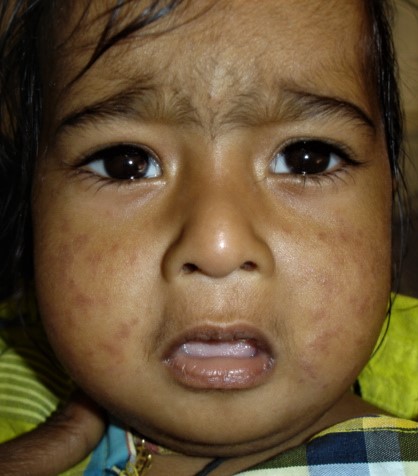
**

1. **Child with scabies rash over body**


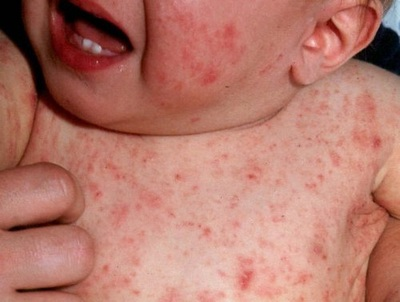
 **
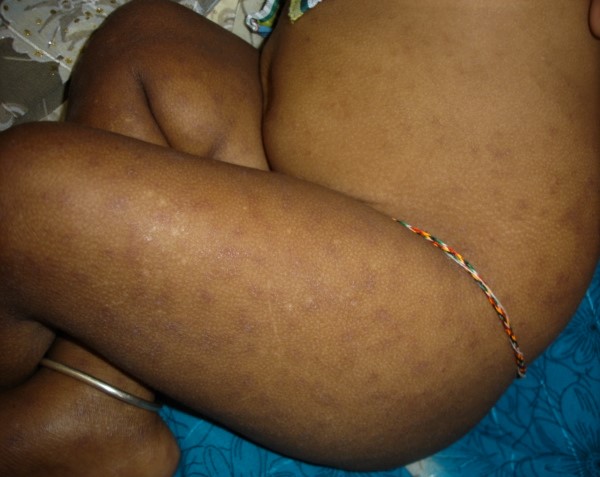
**

1. **Child with scabies rash over hands**

**
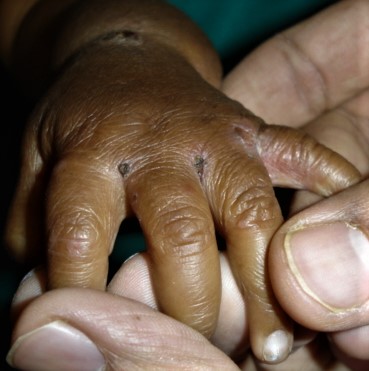

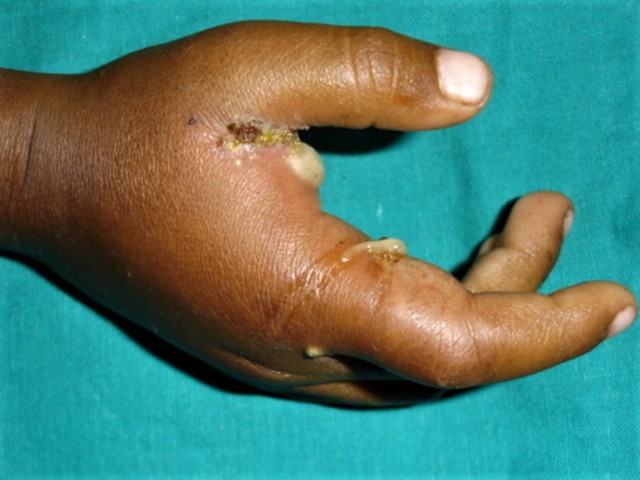
**

**
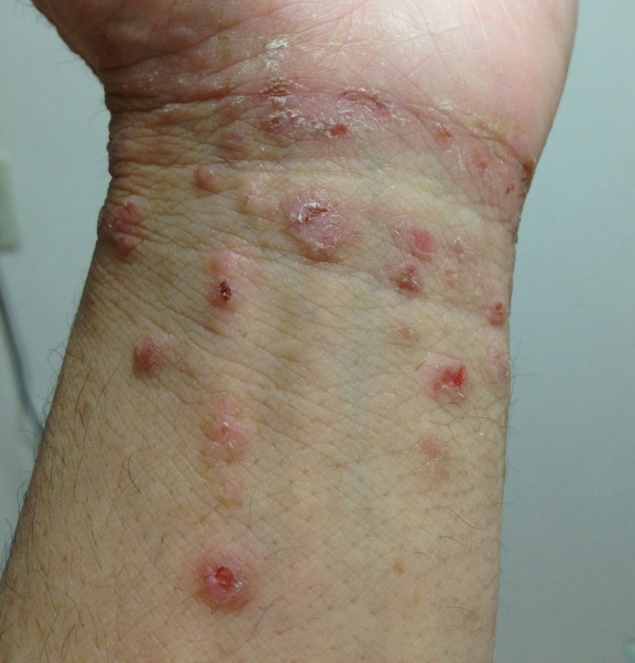
**
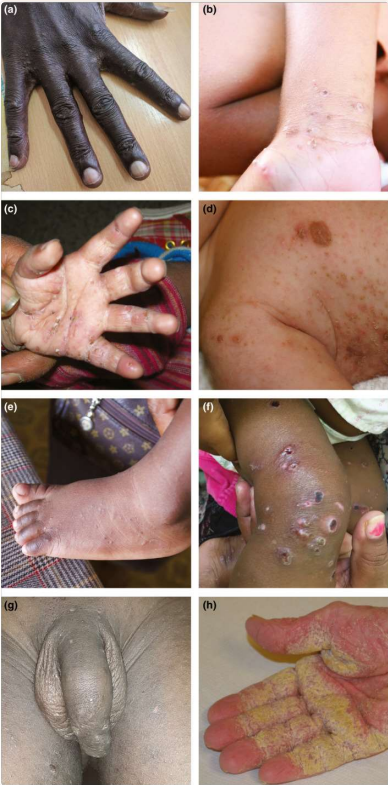


1. **Child with scabies rash over legs**

**
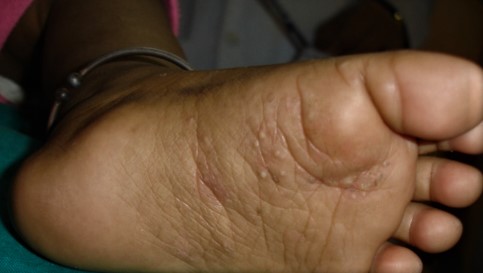
**
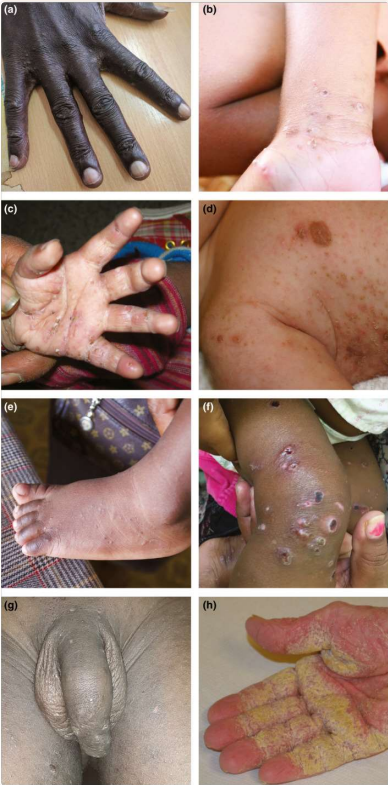


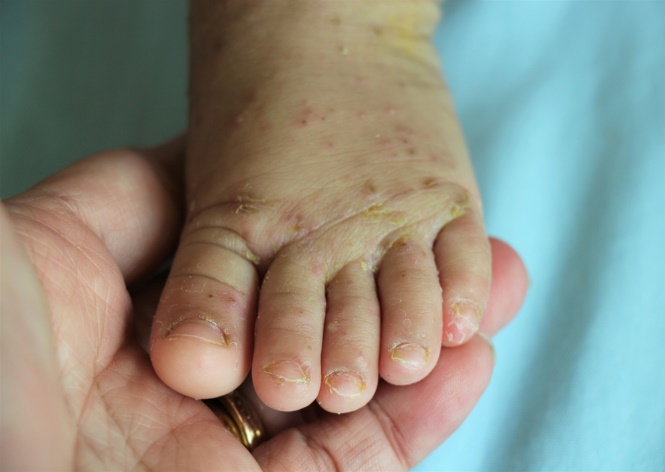
 **
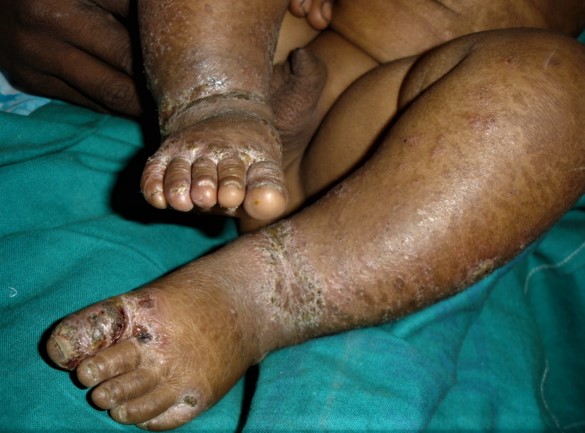
**

1. **Child with scabies lesions on external genital areas**

**
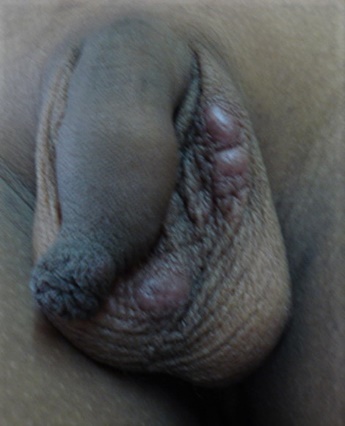

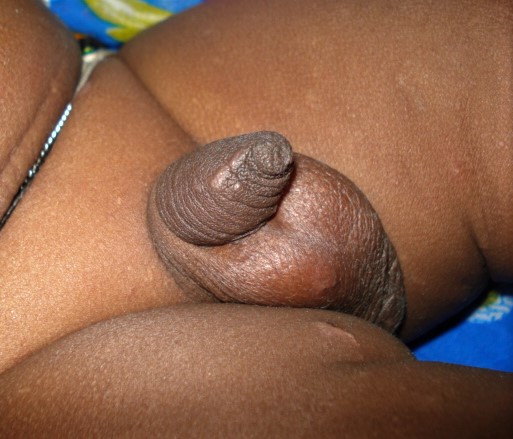
**

**Note:** In children, the most commonly affected sites are in the following order

1. Hands and feet
2. Axilla
3. Face, trunk, arms, legs

**Image credits:**

1. Dr. Aparna Palit, Professor and Head, Department of Dermatology, Venereology and Leprosy, All India Institute of Medical Sciences, Kalyani, West Bengal (Collected from patients with informed verbal consent from parents during out patient visits)
2. Engelman D, Yoshizumi J, Hay RJ, Osti M, Micali G, Norton S et al. The 2020 International Alliance for the Control of Scabies Consensus Criteria for the Diagnosis of Scabies. Br J Dermatol. 2020 Nov;183(5):808-820. doi: 10.1111/bjd.18943. The International Alliance for the Control of Scabies (IACS)

**Text credits:** Parasites – Scabies, Centre for disease control and prevention.
