# Supplementary material for: The impact of training front-line health care workers in improving the yield of scabies diagnosis among children under five in a rural community of West Bengal
Source: PLoS Negl Trop Dis. 2025 Nov 21;19(11):e0013739. doi: 10.1371/journal.pntd.0013739 (PMC12671740; doi:10.1371/journal.pntd.0013739)
Supplement: S2 File — (DOCX) [file pntd.0013739.s003.docx]

**Questionnaire:**

**Project title:** The impact of training front-line health care workers in improving the yield of scabies diagnosis among under five children in a rural community of West Bengal

Serial number:

Date of interview:

Name of the interviewer (ASHA Worker’s Name):

Mobile number of the ASHA Worker:

Village (Lane/Place) name:

Mobile number of the head of household:

**I. Socio-demographic information of parents**

| **Sl no** | **Question** | **Code** | **Mother’s answer** | **Father’s answer** |
| --- | --- | --- | --- | --- |
| 1 | Age (in years) | |  |  |
| 2 | Education status | 1. Illiterate 2. Primary school 3. Middle school 4. High school 5. Higher secondary 6. Graduate/Post-graduate 7. Professional |  |  |
| 3 | Occupation | 1. Working 2. Not-working |  |  |

**II. Socio-demographic information of family**

| **Sl no** | **Question** | **Code** | **Response** |
| --- | --- | --- | --- |
| 1 | Respondent | 1. Mother 2. Father 3. Any other member of family (mention the relationship with child) 4. Care-giver |  |
| 2 | Number of total members in the family | |  |
| 3 | Type of family | 1. Nuclear 2. Extended |  |
| 4 | Religion | 1. Hindu 2. Muslim 3. Christian 4. Others (please specify) |  |
| 5 | Caste | 1. General 2. OBC 3. SC/ST 4. Others (please specify) |  |
| 6 | Monthly family income (in rupees) | |  |
| 7 | Socio-economic status – modified BG prasad scale  Upper, upper middle, middle, lower middle, lower)  (Will be filled by Principal Investigator) | |  |
| 8 | Number of children under five years of age in the family | |  |

**III. Details of under five children in the family**

| **Sl No** | **Question** | **Code** | **Child 1** | **Child 2** | **Child 3** |
| --- | --- | --- | --- | --- | --- |
| 1 | Age (in years and months) | |  |  |  |
| 2 | Gender of the child | 1. Female 2. Male |  |  |  |
| 3 | High risk pregnancy  (from MCP card) | 1. Yes 2. No |  |  |  |
| 4 | Mode of delivery | 1. Normal 2. Assisted/Forceps 3. LSCS |  |  |  |
| 5 | Place of delivery | 1. Government hospital 2. Private hospital/nursing home 3. Home (own) |  |  |  |
| 6 | Pre-term delivery | 1. Yes 2. No |  |  |  |
| 7 | Birth weight (in grams) - from MCP card | |  |  |  |
| 8 | Height of the child (in cms) | |  |  |  |
| 9 | Weight of the child (in kg/grams) | |  |  |  |
| 10 | MUAC of the child (in cms) | |  |  |  |
| 11 | Immunised for age  – from MCP card | 1. Yes 2. No |  |  |  |

**IV.Examination of under five children for scabies**

*(Mark 1 if the answer is ‘Yes’ and mark 2 if the answer is ‘No’)*

| **Sl No** | **Question** | | **Code** | **Child 1** | **Child 2** | **Child 3** |
| --- | --- | --- | --- | --- | --- | --- |
| 1 | Clinical features  (refer to the booklet provided) | 3a: Skin burrows | 1. Yes 2. No |  |  |  |
|  |  | 3b:Typical lesions affecting genitilia (in males) |  |  |  |  |
|  |  | 3c:Typical lesions in typical distribution |  |  |  |  |
|  |  | 3d:Atypical lesions or atypical distribution |  |  |  |  |
|  |  | 3e:Itching |  |  |  |  |
|  |  | 3f:Positive contact history |  |  |  |  |
| 2 | Duration of the symptoms (in days) | | |  |  |  |
| 3 | Diagnosed with scabies by any health care-worker or doctor? | | 1.Yes  2.No |  |  |  |
| 4 | Is any treatment taken for scabies? | | 1.Yes  2.No |  |  |  |
| 5 | If yes, please mention the health facility approached/mode of treatment taken | | 1. Government facility 2. Private practitioner 3. Traditional healer 4. Over the counter 5. Home remedy |  |  |  |
| 6 | If yes, please mention the medications being taken (name of all medications) | | |  |  |  |
| 7 | Overall coding  (Will be done by Principal investigator) | | 1. Clinical scabies 2. Suspected scabies |  |  |  |

1. Clinical scabies: If answer to 3a or 3b or 3c+3e+3f = Yes
2. Suspected scabies: If answer to 3c+3e/3f or 3d+3e+3f = Yes

**V. History of family members:**

| **Sl No** | **Question** | **Code** | **Response** |
| --- | --- | --- | --- |
| 1 | Are you aware of scabies | 1. Yes 2. No |  |
| 2 | If yes, what according to you causes scabies? | |  |
| 3 | Is any of your family member/care-giver diagnosed with scabies at present? | 1. Yes 2. No |  |
| 4 | If yes, mention the number of family members/care-givers affected? | |  |
| 5 | If yes, mention the time of diagnosis (in days) | | **Person 1:** |
|  |  |  | **Person 2:** |
|  |  |  | **Person 3:** |
|  |  |  | **Person 4:** |
| 6 | Was any of your family member/care-giver diagnosed with in the past 6 months? | 1. Yes 2. No |  |
| 7 | If yes, mention the number of family members affected? | |  |
| 8 | If yes, mention the time of diagnosis (in days) | | **Person 1:** |
|  |  |  | **Person 2:** |
|  |  |  | **Person 3:** |
|  |  |  | **Person 4:** |
| 9 | In the past 6 months, did you have any visitor who was diagnosed with/was on treatment for scabies? | 1. Yes 2. No |  |

**VI. Other Environmental details of the family:**

| **Sl No** | **Question** | **Code** | **Response** |
| --- | --- | --- | --- |
| 1 | Water source | 1. Time kol 2. Hand pump 3. Lake/water body 4. Other (Please specify) |  |
| 2 | Adequacy of water supply | 1. Adequate 2. Inadequate |  |
| 3 | Sleeping place | 1. On bed 2. On floor |  |
| 4 | Overcrowding | 1. Present 2. Absent |  |
| 5 | Sharing of linen/clothes | 1. Yes 2. No |  |
| 6 | Frequency of bathing  (Daily, alternate days, twice weekly, weekly, non-specific) | |  |
| 7 | Frequency of washing linen  (Daily, alternate days, twice weekly, weekly, non-specific) | |  |
| 8 | Personal hygiene  (Nails, hair hygiene, teeth, overall appearance) | 1. Good 2. Poor |  |
